# Use of Electronic Vapor Products Before, During, and After Pregnancy Among Women with a Recent Live Birth — Oklahoma and Texas, 2015

**DOI:** 10.15585/mmwr.mm6808a1

**Published:** 2019-03-01

**Authors:** Martha Kapaya, Denise V. D’Angelo, Van T. Tong, Lucinda England, Nan Ruffo, Shanna Cox, Lee Warner, Jennifer Bombard, Tanya Guthrie, Ayesha Lampkins, Brian A. King

**Affiliations:** ^1^Division of Reproductive Health, National Center for Chronic Disease Prevention and Health Promotion, CDC; ^2^Division of Congenital and Developmental Disorders, National Center on Birth Defects and Developmental Disabilities, CDC; ^3^Insignia Federal Group, McLean, Virginia; ^4^Texas Department of State Health Services; ^5^Oklahoma State Department of Health; ^6^Office on Smoking and Health, National Center for Chronic Disease Prevention and Health Promotion, CDC.

Electronic vapor products (EVPs) comprise a diverse group of devices, including electronic cigarettes (e-cigarettes). EVP users inhale an aerosol that typically contains nicotine, flavorings, and other additives (*1*). Nicotine is a developmental toxicant that adversely affects pregnancy and infant outcomes (*2*). Data from the 2015 Pregnancy Risk Assessment Monitoring System (PRAMS) for Oklahoma and Texas were analyzed to estimate population-based EVP use among women with a recent live birth. EVP use before pregnancy (defined as >3 months before pregnancy) and around the time of pregnancy (defined as any time during the 3 months before pregnancy, the last 3 months of pregnancy, or 2–6 months after delivery), reasons for EVP use, and dual use of EVPs and cigarettes were assessed. Prevalence of EVP use was 10.4% before pregnancy and 7.0% around the time of pregnancy, including 1.4% during the last 3 months of pregnancy. Among women using EVPs during the last 3 months of pregnancy, 38.4% reported use of EVPs containing nicotine, and 26.4% were unsure of nicotine content. Among women who had used EVPs and cigarettes, dual use prevalence was 38.0% in the 3 months before pregnancy, 7.7% during the last 3 months of pregnancy, and 11.8% in the 2–6 months after delivery. The most frequently reported reasons for EVP use around the time of pregnancy were curiosity (54.0%), the perception that EVPs might help with quitting or reducing cigarette smoking (45.2%), and the perception of reduced harm to the mother, when compared with cigarette smoking (45.2%). Clear messages that EVP use is not safe during pregnancy are needed, and broad, barrier-free access to evidence-based tobacco cessation strategies need to be made available.

PRAMS is a state- and population-based surveillance system designed to monitor selected self-reported behaviors and experiences before, during, and after pregnancy among women who have had a recent live birth. Participating states select a stratified random sample of women from birth certificate records and survey them by mail 2–6 months after delivery. Women who do not respond to the mailed survey are followed up by telephone.* Oklahoma and Texas included supplementary questions on EVPs on their PRAMS questionnaire in 2015, and data from responses were analyzed for this report. Data were weighted to adjust for noncoverage and nonresponse and represent the total population of women with a live birth in each state in 2015. Weighted response rates were 68% for Oklahoma and 56% for Texas. The sample included 3,277 women, including 1,955 (60%) from Oklahoma and 1,322 (40%) from Texas.

EVP use >3 months before pregnancy was ascertained by counting the number of women who responded affirmatively to the question “Have you ever used electronic vapor products, even one time?” (excluding those who reported use 3 months before, during, and shortly after pregnancy). EVP use around the time of pregnancy was ascertained by responses to questions about three specific time frames: 1) 3 months before pregnancy (“During the 3 months before you got pregnant, on average, how often did you use electronic vapor products?”); 2) during the last 3 months of pregnancy (“During the last 3 months of your pregnancy, on average, how often did you use electronic vapor products?”); and 3) 2–6 months after delivery (at the time the survey was administered) (“Since your new baby was born, on average, how often do you use electronic vapor products that contain nicotine?”). Reasons for EVP use were ascertained from a list of nine options.^†^ Cigarette smoking around the time of pregnancy was assessed among women who reported any cigarette smoking in the past 2 years. Among women who reported having ever used EVPs and having smoked cigarettes in the past 2 years, dual use of EVPs and cigarettes was estimated for each of the three periods.

Weighted prevalence estimates and 95% confidence intervals (CIs) were calculated overall and by state, using SUDAAN (version 11.0, RTI International) to account for the complex sampling design of PRAMS. Chi-squared tests were used to compare differences in the prevalence of EVP use between cigarette smokers and nonsmokers. P-values <0.05 were considered statistically significant.

Overall, among 3,277 women with a recent live birth, 2,533 (82.6%) had never used EVPs; 459 (10.4%), including 15.8% in Oklahoma and 9.7% in Texas, had used EVPs >3 months before pregnancy, but had not used them around the time of pregnancy (Table). The prevalence of EVP use around the time of pregnancy was 7.0% overall (10.3% in Oklahoma and 6.5% in Texas). EVP use during the last 3 months of pregnancy was 1.4% (3.2% in Oklahoma and 1.1% in Texas). Among women who used EVPs during the last three months of pregnancy, 38.4% reported using EVPs containing nicotine, 35.2% reported using EVPs that did not contain nicotine, and 26.4% did not know about the nicotine content of the EVPs they used.

**TABLE:** Weighted prevalence of electronic vapor product (EVP) use and dual use of EVPs and cigarettes among women with a recent live birth (N = 3,277), by timing of use — Pregnancy Risk Assessment Monitoring System, Oklahoma and Texas, 2015

Characteristic (no.)	Timing of EVP use relative to pregnancy											
	>3 months before pregnancy*		Around the time of pregnancy^†^		During 3 months before pregnancy		During last 3 months of pregnancy		2–6 months after delivery		None	
	No.	% (95% CI)	No.	% (95% CI)	No.	% (95% CI)	No.	% (95% CI)	No.	% (95% CI)	No.	% (95% CI)
**Total (3,277)**	**459**	**10.4 (8.7–12.3)**	**285**	**7.0 (5.7–8.6)**	**223**	**5.8 (4.6–7.3)**	**70**	**1.4 (0.9–2.1)**	**96**	**2.1 (1.5–3.1)**	**2,533**	**82.6 (80.3–84.7)**
**State**												
Oklahoma (1,955)	323	15.8 (13.2–18.8)	189	10.3 (8.2–12.9)	142	7.6 (5.8–9.9)	52	3.2 (2.1–5.0)	70	3.5 (2.3–5.2)	1,443	73.8 (70.3–77.1)
Texas (1,322)	136	9.7 (7.9–11.8)	96	6.5 (5.1–8.3)	81	5.6 (4.3–7.3)	18	1.1 (0.6–2.0)	26	2.0 (1.3–3.1)	1,090	83.8 (81.2–86.1)
**Cigarette smoking status^§^**												
Smoker (813)	299	29.8 (24.2–36.2)	219	25.1 (19.8–31.2)	173	21.7 (16.7–27.7)	56	5.1 (3.1–8.2)	73	8.6 (5.6–12.9)	295	45.1 (38.4–51.9)
Nonsmoker (2,428)	159	6.0 (4.6–7.9)	64	2.9 (2.0–4.2)	49	2.3 (1.5–3.4)	13	0.5 (0.2–1.3)	21	0.7 (0.3–1.4)	2,205	91.1 (89.0–92.8)

**Abbreviation:** CI = confidence interval.

* Reported ever using EVPs even one time, but not during the 3 months before pregnancy, the last 3 months of pregnancy, or during the 2–6 months after delivery (i.e., former users).

^†^ Any use within 3 months before pregnancy, during last 3 months of pregnancy, or during the 2–6 months after delivery.

^§^ Any cigarette smoking during the last 2 years.

Prevalence of any cigarette smoking in the past 2 years was 18.5% (813), 16.4% (722) in the 3 months before pregnancy, 6.1% (31) in the last 3 months of pregnancy, and 10.3% (507) during the 2–6 months after delivery. Compared with nonsmokers, a higher proportion of women who smoked cigarettes in the past 2 years used EVPs >3 months before pregnancy (29.8% versus 6.0%; p<0.001) and around the time of pregnancy (25.1% versus 2.9%, p<0.001).

Overall, among women who smoked cigarettes in the past 2 years and had ever used EVPs, use of both cigarettes and EVPs was reported by 38.0% of women during the 3 months before pregnancy, 7.7% during the last 3 months of pregnancy, and 11.8% during the 2–6 months after delivery (Figure 1). The prevalence of EVP use alone was highest during the 2–6 months after delivery (3.8%), and the prevalence of neither cigarette smoking nor EVP use was highest (61.9%) during the last 3 months of pregnancy.

**Figure 1:**
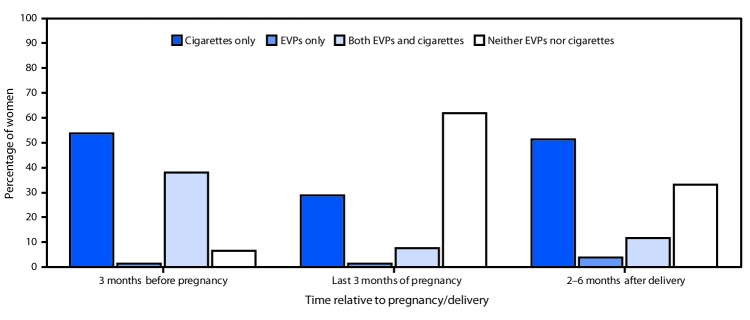
Percentage of women using electronic vapor products (EVPs) and cigarettes 3 months before pregnancy, during the last 3 months of pregnancy, or 2–6 months after delivery, among women with a recent live birth who smoked cigarettes in the last 2 years and ever used EVPs (N = 518) — Pregnancy Risk Assessment Monitoring System, Oklahoma and Texas, 2015

Among women who used EVPs >3 months before pregnancy, the most frequently reported reasons for use were curiosity about the products (78.6%), the perception that EVPs might help with quitting or reducing cigarette smoking (27.4%), the perception that EVPs are less harmful than cigarettes (24.6%), the availability of flavored EVPs (24.5%), and the ability to get EVPs without nicotine (16.9%) (Figure 2). Among women who used EVPs around the time of pregnancy, the most frequently reported reasons for use were curiosity (54.0%), the perception that EVPs might help with quitting or reducing cigarette smoking (45.2%), the perception that EVPs are less harmful to the mother than cigarettes (45.2%), the availability of flavored EVPs (42.3%), and the ability to get EVPs without nicotine (41.4%).

**Figure 2:**
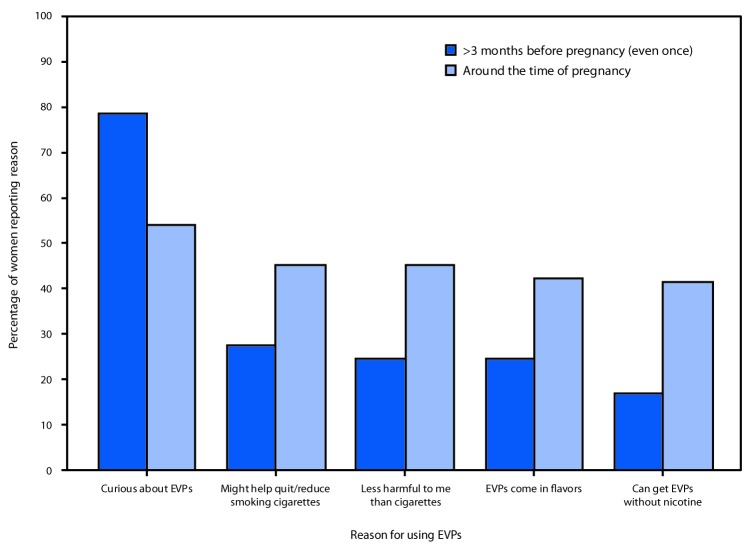
Percentage of women with a recent live birth who reported a reason for using electronic vapor products (EVPs) >3 months before pregnancy (even once) and around the time of pregnancy,* by most frequently reported reasons — Pregnancy Risk Assessment Monitoring System, Oklahoma and Texas, 2015 * Around the time of pregnancy includes 3 months before pregnancy, during last 3 months of pregnancy, or 2–6 months after delivery.

## Discussion

These findings build on prior studies assessing use of tobacco products, including EVPs, among pregnant women (*3*–*5*) by highlighting the prevalence of EVP use, reasons for EVP use, and dual cigarette and EVP use in a large population-based sample. The current study confirms that, although EVPs are not safe to use during pregnancy (*1*,*2*), 7.0% of women (approximately one in 15 women) in Oklahoma and Texas who had a recent live birth used EVPs around the time of pregnancy; moreover, EVP use was higher among women who had smoked cigarettes in the past 2 years. Among women who smoked cigarettes in the past 2 years and had ever used EVPs, dual use of EVPs and cigarettes was higher in the 3 months before pregnancy and lower during the last 3 months of pregnancy and the 2–6 months after delivery.

This study’s findings that 10% of women with a recent live birth used EVPs >3 months before becoming pregnant and 1.4% used them during the last 3 months of pregnancy differ from findings of the Population Assessment of Tobacco and Health (PATH) Study, which found that nearly twice as many (18.4%) pregnant women were former e-cigarettes users, and 4.9% reported use during pregnancy (*3*). The prevalence in the current study was likely lower because the PATH Study, a large nationally representative household-based study, assessed use during the entire pregnancy, rather than the last 3 months. Nevertheless, both studies found that a higher proportion of cigarette smokers used EVPs than did nonsmokers.

Nearly half of women who used EVPs around the time of pregnancy (45.2%) reported using the products because they perceived EVPs to be less harmful to them than regular cigarettes or that EVPs would help them with quitting or reducing smoking. Notably, the proportion of these users was approximately twice that of those who had used EVPs >3 months before pregnancy (27.4%). This suggests that women are aware of the harms of smoking during pregnancy, and, perceiving EVPs to be a safer alternative during pregnancy, might be using EVPs to mitigate those harms. This finding was consistent with an Internet survey of perceptions and prevalence of e-cigarette use among 445 pregnant women: among 67 pregnant women who reported using cigarettes or e-cigarettes, 50 (74.6%) reported switching from cigarettes to e-cigarettes or beginning use of e-cigarettes upon learning they were pregnant (*4*). Among those who switched, 23 (46%) reported that they believed e-cigarettes were safer for them or their child than cigarettes, and nine (18%) reported switching to quit smoking cigarettes (*4*). A smaller clinical trial assessing smoking cessation among 103 pregnant smokers found that a similar proportion of women (14%) reported using e-cigarettes for smoking cessation during pregnancy (*5*).

Although aerosol from EVPs contains lower levels of toxicants than does cigarette smoke (*1*,*6*), EVPs are not safe to use during pregnancy because most contain nicotine (*7*). Nicotine, a developmental toxicant, adversely affects pregnancy and infant outcomes (*2*). Although the U.S. Preventive Services Task Force has determined that, currently, insufficient evidence exists to recommend EVPs for tobacco cessation among adults (including pregnant women) (*8*), many women report using EVPs in an attempt to quit smoking cigarettes around the time of pregnancy (*4*,*5*).

Barrier-free smoking cessation strategies with established effectiveness and safety need to be made available to all pregnant women (*2*). Behavioral intervention is a first-line treatment to help pregnant women quit smoking (*2*,*8*). In addition, Food and Drug Administration–recommended pharmacotherapy products (including nicotine replacement therapy), can be considered during pregnancy with close supervision of a clinician (*2*,*8*); these products don’t contain the other harmful substances that have been found in the aerosol emitted from EVPs (*1*,*6*). However, variation in coverage provided by health insurance payers might prohibit access to effective treatment. In Texas, for example, women with Medicaid coverage have access to the full range of cessation interventions, with the exception of group and individual counseling, for which coverage varies by plan. In Oklahoma, Medicaid covers all treatment options except group counseling.^§^ In addition, the Oklahoma Tobacco Helpline (1-800-QUIT NOW), a statewide, free, 24/7 tobacco cessation helpline, offers various options to aid in cessation efforts. In Texas, the toll-free Quitline phone number, 1-877-YES-QUIT was part of the resource list provided to mothers selected for the 2015 PRAMS survey. In both Oklahoma and Texas, all plans in the Health Insurance Marketplace are required to cover tobacco cessation treatment; however, specific coverage varies by plan. In both states, private insurance plans are not required to cover cessation treatment, which could limit options available to some women.

The findings in this report are subject to at least three limitations. First, because data were self-reported postpartum, they are subject to recall and social desirability biases, which might result in underestimates of EVP use and cigarette smoking. Second, these data are only representative of women with a recent live birth in Oklahoma and Texas. Finally, because EVPs are an emerging product, these point-in-time estimates from 2015 might not reflect trends in use in Oklahoma and Texas in more recent years, including the use of increasingly popular EVPs shaped like USB flash drives, including JUUL, that contain very high levels of nicotine (*9*).

Among women with a recent live birth, many reported use of EVPs. Moreover, among those who used EVPs, a substantial percentage used EVPs in an attempt to quit smoking cigarettes, suggesting a possible lack of awareness of, or access to, evidence-based approaches to smoking cessation. Messages that EVPs are not safe to use during pregnancy and that nicotine adversely affects fetal development and infant outcomes need to be clearly communicated. Health care providers can offer education, counseling, and evidence-based cessation treatment to prevent use of all tobacco products, including EVPs, by women before, during, and after pregnancy.

**Summary**

**What is already known about this topic?**

Most electronic vapor products (EVPs) contain nicotine, a developmental toxicant, and other harmful additives.

**What is added by this report?**

In 2015, 7.0% of women with a recent live birth in Oklahoma and Texas reported using EVPs shortly before, during, or after pregnancy, with 1.4% reporting use during pregnancy. Among prenatal EVP users, 38.4% reported using EVPs containing nicotine, and 26.4% did not know if the EVPs they used contained nicotine.

**What are the implications for public health practice?**

Messages that EVPs are not safe to use during pregnancy need to be clearly communicated. Education, counseling, and evidence-based cessation treatment could assist reproductive-aged women in preventing or reducing the use of all tobacco products, including EVPs.

## Footnotes

* https://www.cdc.gov/PRAMS.

† Assessed reasons included the following: they cost less than cigarettes or other forms of tobacco; EVPs can be used where smoking is not allowed; I can get EVPs without nicotine; they might be less harmful to me than regular cigarettes; they might be less harmful to my baby than regular cigarettes; they might be less harmful to the people around me than regular cigarettes; EVPs come in flavors; EVPs might help me quit or reduce smoking regular cigarettes; I was curious about EVPs.

§ http//www.lungusa2.org/cessation2/.
